# Twenty-four hour variability of inverted T-waves in patients with apical hypertrophic cardiomyopathy

**DOI:** 10.3389/fcvm.2022.1004178

**Published:** 2022-09-21

**Authors:** Fei Ma, Yating Yang, Jingwen Tao, Xiaoyan Deng, Xufeng Chen, Jingjing Fan, Xuelei Bai, Tongyu Dai, Sheng Li, Xiaoyun Yang, Fan Lin

**Affiliations:** ^1^Division of Cardiology, Department of Internal Medicine, Tongji Hospital, Tongji Medical College, Huazhong University of Science and Technology, Wuhan, China; ^2^Hubei Key Laboratory of Genetics and Molecular Mechanisms of Cardiological Disorders, Huazhong University of Science and Technology, Wuhan, China; ^3^Laboratory of Cardiac Physiology, Nanyang Second General Hospital, Nanyang, China; ^4^Department of Electrophysiology, Changde First People’s Hospital, Changde, China

**Keywords:** electrocardiogram, T-wave amplitudes, apical hypertrophic cardiomyopathy, coronary artery disease, normalization of abnormal T-waves

## Abstract

**Background:**

Patients with apical hypertrophic cardiomyopathy (ApHCM) have marked inverted T-waves that vary over several years. Inverted T-waves in ApHCM are unstable, but it is unclear whether this change is due to coronary artery disease (CAD) or if it is a characteristic of ApHCM itself. We aimed to study the characteristics of inverted T-waves in patients with ApHCM over the course of 24 h to improve the diagnostic indices of ApHCM.

**Methods:**

We examined 83 patients with ApHCM and 89 patients with CAD (who served as the control group). All patients underwent a 24-h dynamic electrocardiogram (ECG). We analyzed the average depth of inverted T-waves per minute and sorted them from shallow to deep; the sorted ECG segments at the 10^th^, 50^th^, and 90^th^ positions of the T-wave were subsequently analyzed.

**Results:**

The amplitudes of inverted T-waves in ApHCM corresponding to the 10^th^, 50^th^, and 90^th^ percentiles were −5.13 ± 4.11, −8.10 ± 4.55, and −10.9 ± 5.04 mm, respectively. Changes in the degree of inverted T-waves were greater in ApHCM than in CAD. T-wave amplitudes in ApHCM were strongly associated with heart rate and circadian rhythm and only weakly associated with CAD and posture. Maximum T-wave amplitudes in the CAD group were <10 mm, while 68% of patients with ApHCM had maximum T-wave amplitudes >10 mm, and all patients with ApHCM aged <50 years had maximum T-wave amplitudes >10 mm.

**Conclusion:**

Notable variations in the T-waves of patients with ApHCM were observed over 24 h. ECG examinations during states of inactivity (comparable to sleep) improved the sensitivity of the diagnosis of ApHCM. Inverted T-wave amplitudes correlated with heart rate and circadian rhythm, where T-wave changes in ApHCM may be due to the normalization of abnormal T-waves effect. Identifying T-wave amplitudes >10 mm can effectively improve the diagnostic rate of ApHCM, especially in patients aged <50 years. The short-term change in T-waves in patients with ApHCM could serve as a novel index that will help in the diagnosis of ApHCM.

## Introduction

Apical hypertrophic cardiomyopathy (ApHCM) is characterized by myocardial hypertrophy, predominantly in the left ventricular (LV) apex, with “giant” negative T-waves (>10 mm) observed in the left precordial leads of an electrocardiogram (ECG) (1). ApHCM is an uncommon phenotype of hypertrophic cardiomyopathy (HCM) (2). In North America, ApHCM accounts for 3% of HCM cases; in Japan and China, these figures are as high as 15 and 16%, respectively (3, 4). One-third of ApHCM patients may develop unfavorable clinical events and potentially life-threatening complications, such as myocardial infarction, arrhythmias, and stroke (5). However, the misdiagnosis rate of ApHCM is high and easily confused with coronary artery disease (CAD) (6).

There are no official recommendations to guide ApHCM diagnosis (7). The ultrasound sensitivity for ApHCM diagnosis is not high, and magnetic resonance imaging (MRI) is not a routine screening method in this field (8). The diagnosis of ApHCM using ECG is based on the T-wave depth (9), although the T wave is unstable in both the short- and long-term. ECG abnormalities of ApHCM, including a high R-wave amplitude and marked T-wave inversion, were present before imaging could demonstrate LV hypertrophy (10). Koga et al. indicated that T-wave negativity in patients with ApHCM increased in the intermediate follow-up group and disappeared in 71% of patients in the long-term follow-up group (5, 11). The interval within which T-wave changes occur in patients with ApHCM is reportedly 1–5 years (12, 13); however, reports by Kereiakes et al. (14), Alfonso et al. (15), and Fujii et al. (16) have these intervals as ranging from 2 to 5 days.

Despite this, the characteristics of short-term inverted T-waves in patients with ApHCM have not been fully studied. Clinically, when inverted T-waves are recorded during ECG examinations, patients with ApHCM who have chest pain are suspected of having acute coronary syndrome (6). Therefore, this study aimed to describe the variations in inverted T-waves in patients with ApHCM, over 24 h and how these T-waves differ from those in patients with CAD. In doing so, we aimed to obtain a more sensitive diagnostic method for ApHCM.

## Materials and methods

### Patients

We included 83 patients with ApHCM (diagnosed by MRI or ultrasound) from Tongji hospital who underwent 24-h dynamic electrocardiogram (DCG) and coronary angiography between February 2016 and February 2020. Eighty-nine patients with CAD and inverted T-waves in the left precordial lead were included as controls. All patients with CAD were diagnosed using coronary angiography, and at least one coronary artery was narrowed to over 80%.

Patients with myocardial infarction were excluded from the ApHCM and CAD study groups. The study was performed retrospectively, where clinical data were identified prior to analyses. Patient identity remained anonymous. Due to the observational nature of the study, Tongji Hospital provided ethical approval and waived the requirement for informed consent.

### Diagnostic criteria

Diagnostic criteria for ApHCM included asymmetrical LV hypertrophy confined predominantly to the LV apex, with an apical wall thickness of 15 mm and a maximal apical-to-posterior wall thickness ratio of 1.5 (based on two-dimensional ultrasound or MRI) (5).

### Echocardiography

Doppler echo studies were performed using commercially available ultrasound equipment. Left atrial dimension at end-systole, standard LV dimensions at end-diastole and end-systole, and LV wall thickness were obtained from two-dimensional imaging, according to the standards of the American Society of Echocardiography (17). Maximum apical wall thickness was obtained from standard apical four- and two-chamber views at end-diastole (5). The diagnostic criteria for ApHCM include asymmetric LV hypertrophy (confined predominantly to the LV apex), with an apical wall thickness ≥15 mm and a maximal apical-to-posterior wall thickness ratio ≥1.5 (assessed using ultrasonography or MRI) (5).

### Electrocardiographic analysis

All the patients underwent 24-h DCG. “Giant” T-wave negativity was defined as a negative T-wave voltage of 1 mV (10 mm) in any lead (5). T-wave amplitude was measured in the left precordial lead of maximal negativity (lead V4 or V5), and R-wave amplitude was measured in the left precordial lead, at the initial examination. ECGs were recorded at a sampling rate of 500 Hz, using GE-Marquette type 3500 or 5500 ECG machines (GE Healthcare, Milwaukee, WI, USA). The 24-h dynamic 12-lead ECGs were recorded by a Holter machine (DMS Holter Company, Stateline, NV, USA). After setup, the Holter Analysis System provided the average value of T-wave depth or height, per minute, with the corresponding time.

### Analysis of negative T-wave depth

We analyzed the average depths of inverted T-waves per minute in the left precordial lead and sorted them from shallow to deep. Next, fragments corresponding to the 10^th^, 50^th^, and 90^th^ periods were selected for further analysis. ECG fragments corresponding to maximum inverted T-wave amplitudes were also selected for further research. ECG fragments corresponding to maximum heart rate (HR_*Max*_), minimum heart rate (HR_*Min*_), and average heart rate (HR_*Avg*_) were also selected for analysis. To analyze the difference between day and night T-waves, three data distribution intervals of 10^th^, 50^th^ and 90^th^ percentile data (corresponding intervals: 7.5–12.5, 47.5–52.5, and 87.5–92.5%, using the 10^th^, 50^th^ and 90^th^ percentiles as the center of corresponding intervals) were selected to analyze the data distribution probability corresponding to different periods of the day. We hypothetically divided 24 h into three periods—from 23:00 to 07:00 (first day-time period), 07:00 to 15:00 (second day-time period), and 15:00 to 23:00 (third day-time period)—to analyze subsequent periods of average T-waves in different percentiles.

### T-waves from different percentiles and the diagnosis of apical hypertrophic cardiomyopathy

Inverted T-waves (in all except the III, aVR, and V1 leads) are highly suggestive of structural or ischemic heart disease (18). Over 24-h, we mixed 249 (10 s) ECG fragments from the ApHCM group and 267 ECG (10 s) fragments from the CAD (control) group, with fragments corresponding to the 10^th^, 50^th^, and 90^th^ percentiles for each patient. These 516 ECG fragments were randomly sorted and submitted for evaluation by three cardiologists, all of whom had worked in their field for >10 years. ECGs indicating ApHCM or myocardial ischemia, as determined by two or more physicians, ensured consistency of the diagnosis. In terms of results, we used a minus sign (−) to indicate that the doctor did not diagnose ApHCM, and a plus sign (+) to indicate that the doctor did diagnose ApHCM.

### Statistics

Continuous data are expressed as mean ± SD, unless otherwise specified. Continuous variables were compared using two-tailed unpaired and paired Student’s *t*-tests, Chi-square tests, and Wilcoxon signed-rank tests. Statistical significance was regarded as *P* < 0.05. Data analysis was performed using SPSS 19.0 and GraphPad Prism 8.

## Results

The characteristics of patients in the ApHCM and CAD groups are shown in Table 1. No statistical differences in age (58.03 ± 14.32 vs. 58.34 ± 10.93 years) or sex (80% men vs. 69.7% women) were found between the ApHCM and CAD groups. Chest pain at clinical presentation was more frequent in the CAD group than in the ApHCM group (94.3 vs. 40.9%). Thicker compartments and apical segments and larger left atria were more frequently observed in the ApHCM group than in the CAD group. Coronary angiography data are also summarized in Table 1. In the ApHCM group, 36 (43.4%) patients had negative coronary angiography, 32 (38.5%) had coronary stenosis, and 15 (18.1%) had myocardial bridging. In the CAD group, 89 (100%) patients had coronary stenosis.

**TABLE 1:** Baseline characteristics of the study population.

Parameters	ApHCM (*N* = 83)	CAD (*N* = 89)	*P*-value
Male, *n* (%)	67 (80.7%)	62 (69.7%)	0.114
Age, mean ± SD	58.03 ± 14.32	58.34 ± 10.93	0.872
**Symptoms, *n* (%)**			
Chest pain	34 (40.9%)	84 (94.3%)	<0.001
Dyspnea	2 (2.4%)	2 (2.2%)	1
Palpitations	7 (8.4%)	1 (1.1%)	0.065
Other symptoms	40 (48.1%)	2 (2.2%)	<0.001
**Echocardiograph, mean ± SD**			
LAD (mm)	38.04 ± 6.88	34.85 ± 6.29	0.001
LVEDd (mm)	48.76 ± 4.31	47.97 ± 5.23	0.288
LVEF (%)	59.12 ± 12.30	61.57 ± 8.54	0.133
IVS (mm)	12.14 ± 3.13	10.12 ± 1.18	<0.001
LVPW (mm)	9.65 ± 1.73	9.83 ± 0.97	0.395
LV apex (mm)	17.77 ± 1.63	9.58 ± 0.69	<0.001
**Coronary angiography, *n* (%)**			
Negative	36 (43.4%)	0 (0%)	<0.001
Coronary artery lesions	32 (38.5%)	89 (100%)	<0.001
Myocardial bridges	15 (18.1%)	0 (0%)	<0.001

ApHCM, apical hypertrophic cardiomyopathy; CAD, coronary artery disease; SD, standard deviation; LAD, left atrial diameter; LVEF, left ventricular ejection fraction; LVEDd, left ventricular end-diastolic dimensions; IVS, interventricular septum; LVPW, posterior wall of left ventricle; LV apex, left ventricular apex.

### Analysis of inverted T-waves, per minute, within 24 h

The amplitudes of negative T-waves in the ApHCM group corresponding to the 10^th^, 50^th^, and 90^th^ percentiles were −5.13 ± 4.11, −8.10 ± 4.55, and −10.9 ± 5.04 mm, respectively. The amplitudes of negative T-waves in the CAD group were −1.21 ± 1.27, −2.17 ± 1.47, and −3.18 ± 1.8 mm, respectively (Table 2).

**TABLE 2:** Negative T-wave amplitudes corresponding to the 10^th^, 50^th^, and 90^th^ percentiles compared with HR_Max_, HR_Min_, and HR_Avg_ T-waves.

	ApHCM (*N* = 83)		CAD (*N* = 89)	
	HR (bpm)	T-wave (mm)	HR (bpm)	T-wave (mm)
T-wave of HR_max_	108.43 ± 19.80	−5.53 ± 4.16*	106.94 ± 15.7	−1.30 ± 1.23
T-wave of HR_average_	66.60 ± 11.19	−8.02 ± 4.74*	69.35 ± 9.28	−2.25 ± 1.53
T-wave of HR_min_	47.89 ± 7.41	−10.01 ± 5.35*	47.9 ± 7.88	−2.59 ± 1.83
T-wave-10^th^	76.48 ± 10.95	−5.13 ± 4.11*	72.39 ± 10.45	−1.21 ± 1.27
T-wave-50^th^	67.09 ± 8.32	−8.10 ± 4.55*	68.95 ± 9.14	−2.17 ± 1.47
T-wave-90^th^	62.00 ± 8.20	−10.90 ± 5.04*	66.34 ± 10.01	−3.18 ± 1.81
△T_HRmax–HRmin_	30.54 ± 15.78	4.48 ± 3.05*	28.95 ± 8.97	1.28 ± 1.56
△T10^th^−90^th^	14.48 ± 9.48^#^	5.79 ± 2.75*	6.05 ± 8.48	1.83 ± 1.48

All data are represented as mean ± SD.

*P < 0.001, T-wave of ApHCM group when compared with CAD group.

^#^*P* < 0.001, heart rate of ApHCM group when compared with CAD group.

△T10^th^−90^th^ = T-wave-10^th^ − T-wave-90^th^; △T_HRmax–HRmi*n*_ = T_HRmax_ − T_HRmin_.

ApHCM, apical hypertrophic cardiomyopathy; CAD, coronary artery disease; BPM, beats per minute; HR, heart rate.

We analyzed the characteristics of T-waves in different percentiles for all patients. Heart rates of patients in the ApHCM group, corresponding to T-waves at the 10^th^, 50^th^, and 90^th^ percentiles (T-wave-10^th^, T-wave-50^th^, and T-wave-90^th^), were 76.48 ± 10.95, 67.09 ± 8.32, and 62.00 ± 8.20 beats per minute (bpm), respectively. Heart rates of patients in the CAD group, corresponding to T-wave-10^th^, T-wave-50^th^, and T-wave-90^th^, were 72.39 ± 10.45, 68.95 ± 9.14, and 66.34 ± 10.01 bpm, respectively (Table 2). Compared with T-wave-10^th^ heart rates, T-wave-50^th^ and 90^th^ heart rates were significantly different between the ApHCM and CAD groups.

In the ApHCM group, heart rates and T-waves corresponding to HR_*Max*_ were 108.43 ± 19.80 bpm and −5.53 ± 4.16 mm, respectively, and heart rates and T-waves corresponding to T-wave-10^th^ were 76.48 ± 10.95 bpm and −5.13 ± 4.11 mm, respectively. Heart rates corresponding to HR_*Min*_ were slower than those corresponding to T-wave-90^th^, while the amplitude of T-waves corresponding to T-wave-90^th^ was deeper than those corresponding to HR_*Max*_. Heart rates and T-waves corresponding to HR_*Min*_ were 47.89 ± 7.41 bpm and −10.01 ± 5.35 mm, respectively, and heart rates and T-waves corresponding to T-wave-90^th^ were 62 ± 8.20 bpm and −10.9 ± 5.04 mm, respectively. Heart rates corresponding to HR_*Min*_ were slower than those corresponding to T-wave-90^th^, while the amplitude of T-waves corresponding to T-wave-90^th^ was deeper than those corresponding to HR_*Max*_. In the ApHCM group, T-wave amplitudes were closely related to heart rate; however, the slowest heart rate did not correspond to the deepest T-wave, and the fastest heart rate did not correspond to the shallowest T-wave. These observations were also found in the CAD group.

△T10^th^−90^th^ and △T_*HRmax–HRmin*_ can reflect the changing amplitude of T-waves. In the ApHCM group, △T10^th^−90^th^ was 5.79 ± 2.75 mm and △T_*HRmax–HRmin*_ was 4.48 ± 3.05 mm, significantly higher than that in the CAD group—1.83 ± 1.48 and 1.28 ± 1.56 mm, respectively.

### Analysis of T-wave distribution characteristics in patients with apical hypertrophic cardiomyopathy during different periods of the day

**FIGURE 1:**
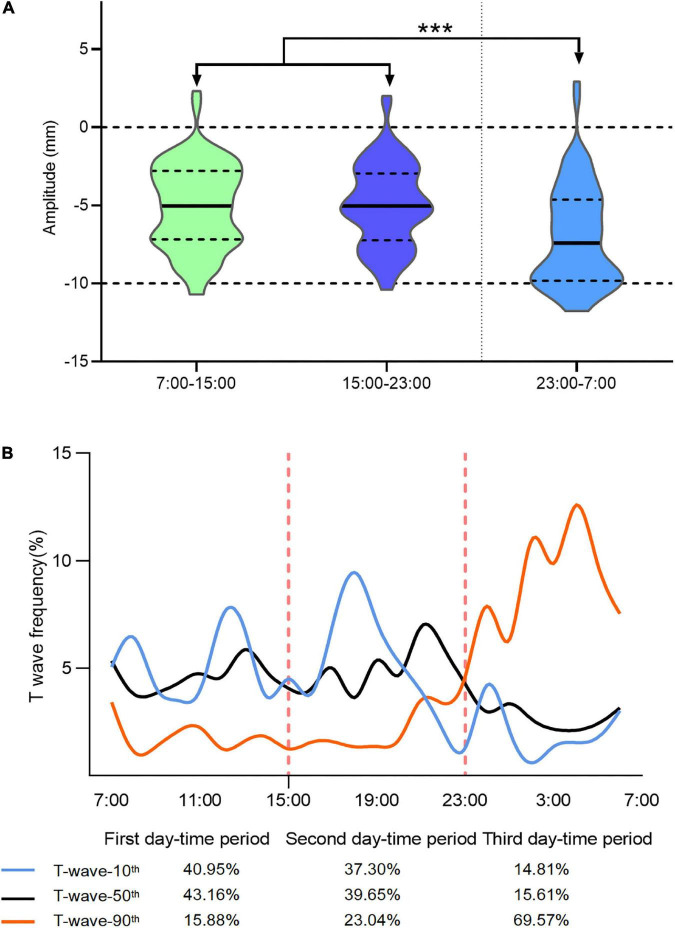
Analysis of T-wave distribution characteristics at different periods of the day. The average T-wave amplitude from 07:00 to 15:00 (the first day-time period) was –7.20 ± 4.43 mm and that from 15:00 to 23:00 (the second day-time period) hours was –7.13 ± 4.54 mm; the amplitude of T-waves were similar in the first and second day-time periods. The average T-wave amplitude from 15:00 to 23:00 (the third day-time period) was –9.00 ± 4.68 mm, which was significantly increased from the T-wave amplitudes recorded in the first and second day-time periods (***P < 0.0001; **A**). The distribution probabilities of ECGs corresponding to the 10^th^, 50^th^, and 90^th^ percentile T-waves in different time periods of a day **(B)**.

The mean T-wave amplitude in the first and second day-time periods was −7.20 ± 4.43 and −7.13 ± 4.54 mm, respectively; the differences between the two time periods were not statistically significant. However, the mean T-wave amplitude in the third day-time period was −9.00 ± 4.68 mm, which was significantly different from that in the first and second day-time periods (*P*-value < 0.001; Figure 1A).

We calculated ECG distribution probabilities corresponding to the 10^th^, 50^th^, and 90^th^ percentile T-waves for the different day-time periods. Regarding the 10^th^ percentile T-waves, 40.95, 37.3, and 14.81% were in the first, second, and third day-time periods, respectively. Similarly, regarding the 50^th^ percentile T-waves, 43.16, 39.65, and 15.61% were in the first, second, and third day-time periods, respectively. Finally, regarding the 90^th^ percentile T-waves, 15.88, 23.04, and 69.57% were in the first, second, and third day-time period, respectively.

Between T-wave-90^th^ to T-wave-10^th^ and T-wave-50^th^, a circadian rhythm was observed. Most of T-wave-90^th^ appeared between 23:00 and 7:00 h, while the T-waves in the T-wave-10^th^ rarely appeared in this period (Figure 1B). The curve corresponding to T-wave-10^th^ started at 21:00, which is a time that is lower than the average probability of the first and second day-time periods. The curve probability corresponding to T-wave-90^th^ between 20:00 and 08:00 h was higher than that corresponding to the other periods; no difference in the distribution probability of the curve corresponding to T-wave-50^th^ throughout the day was found. Meanwhile, the T-wave distribution characteristics in patients with CAD during different periods of the day also exhibit certain cardiac rhythm fluctuations, although the degree of change is not as obvious as that of ApHCM (Supplementary Figure 1).

### Subgroup analysis of apical hypertrophic cardiomyopathy: Patients with apical hypertrophic cardiomyopathy who did or did not have coronary artery disease

All 83 patients with ApHCM included in this study underwent coronary angiography (Table 3); 36 of them had negative coronary angiography results (subgroup A), 32 had coronary lesions (subgroup B), and 15 had myocardial bridges (subgroup C). Comparing the depth differences of inverted T-waves in the 10^th^, 50^th^, and 90^th^ positions of patients in subgroups A and B, the *P*-values were 0.49, 0.37, and 0.22, respectively. Comparing the depth differences of inverted T-waves in the 10^th^, 50^th^, and 90^th^ positions of patients in subgroups A and C, the *P*-values were 0.10, 0.21, and 0.42, respectively.

**TABLE 3:** Relationship between T-wave changes and coronary artery lesions in patients with ApHCM.

	Negative, *n* = 36 (mm)	Coronary artery lesions, *n* = 32 (mm)	*P*-value	Myocardial bridges, *n* = 15 (mm)	*P*-value
T-wave-10^th^	−4.7 ± 4.60	−4.7 ± 3.48	0.49	−7.56 ± 5.99	0.10
T-wave-50^th^	−8.25 ± 5.43	−7.68 ± 4.23	0.37	−10.23 ± 6.09	0.21
T-wave-90^th^	−11.6 ± 5.96	−10.14 ± 4.71	0.22	−12.12 ± 6.07	0.42

All data are represented as mean ± SD.

### Analysis of apical hypertrophic cardiomyopathy diagnosis by inverted T-waves with different amplitudes

**FIGURE 2:**
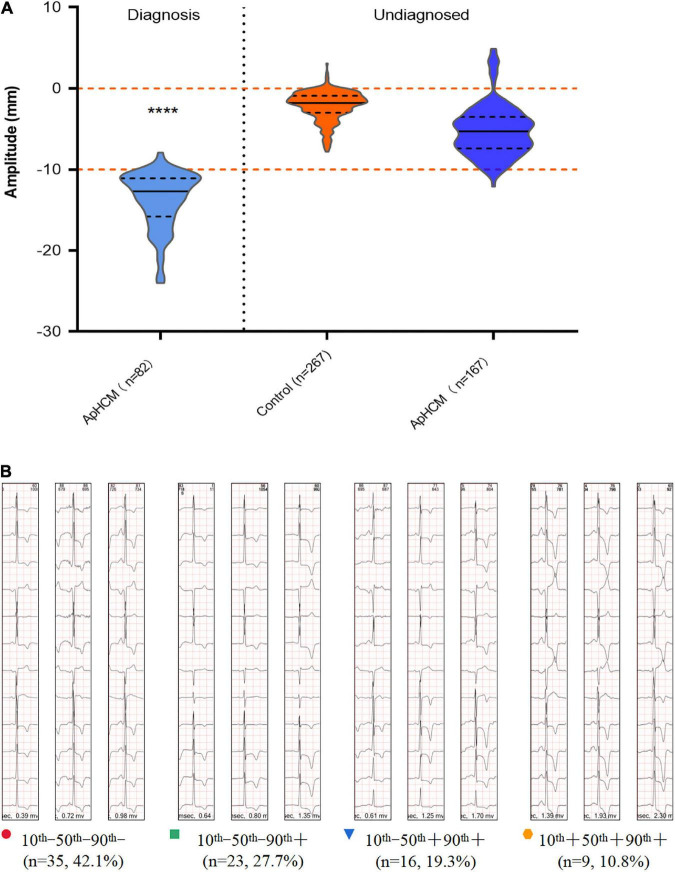
Diagnostic significance of T-wave depth in ApHCM. **(A)** The relationship between T-wave depth and the diagnosis of ApHCM in patients with ApHCM compared with patients with CAD (****P < 0.0001). A total of 167 ECGs are not diagnosed with ApHCM, while 82 ECGs are diagnosed with ApHCM. Diagnosis of different percentile T-waves in each patient with ApHCM was performed by cardiologists. **(B)** Results were divided into four types, each of which corresponded to the number of cases and the proportion of each type. The minus sign (–) indicates that the doctor did not diagnose ApHCM, and a plus sign (+) indicates a diagnosis of ApHCM.

All 267 ECG fragments of patients with CAD were not diagnosed with ApHCM. Of the 249 ECG fragments of patients with ApHCM, 82 were diagnosed with ApHCM, while 167 were not (Figure 2A). However, the percentage of negative T-waves with amplitudes >10 mm in undiagnosed ECGs fragments was 2.99%, while the percentage of diagnosed ECGs fragments with an amplitude >10 mm was 97.56%. Figure 2B shows the diagnosis of ApHCM in different ECG percentiles for each patient. Of the 83 patients, 35 (42.1%) (10^th^− 50^th^− 90^th^−) were not diagnosed with ApHCM at the 10^th^, 50^th^, and 90^th^ percentiles and 23 (27.7%) (10^th^− 50^th^− 90^th^ +) were not diagnosed with ApHCM at the 10^th^ and 50^th^ percentiles but were diagnosed with ApHCM at the 90^th^ percentile. Sixteen (19.3%) patients (10^th^− 50^th^ + 90^th^ +) were not diagnosed with ApHCM at the 10^th^ percentile but were diagnosed at the 50^th^ and 90^th^ percentile. Only nine (10.8%) patients (10^th^ + 50^th^ + 90^th^ +) were diagnosed with ApHCM at the 10^th^, 50^th^, and 90^th^ percentiles. Approximately 46% of patients (10^th^− 50^th^− 90^th^ + and 10^th^− 50^th^ + 90^th^ +) had different results in the diagnosis of ApHCM at different T-wave levels.

**FIGURE 3:**
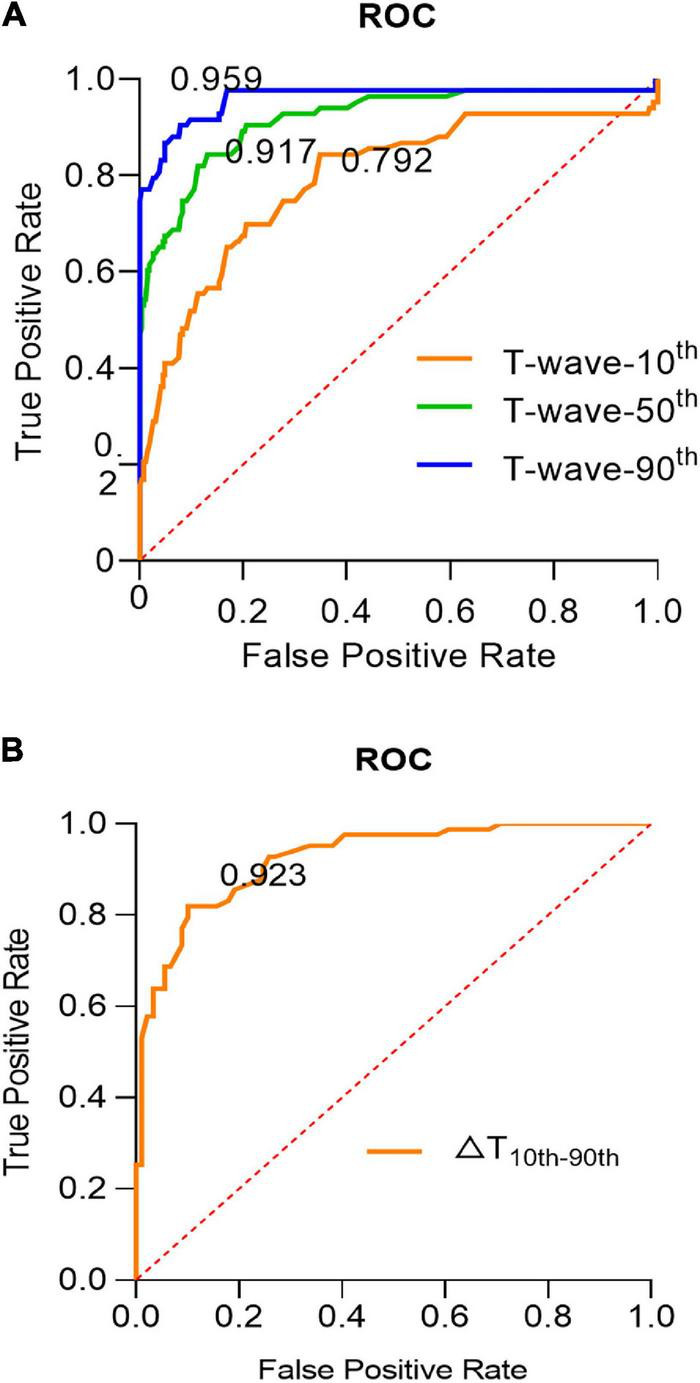
The influence of the different T-waves’ percentiles on detection performance. **(A)** The ROC curves for the effect of different percentile T-waves on detection performance, the yellow, green, and blue lines, respectively, represent the T-wave-10^th^, T-wave-50^th^, and T-wave-90^th^. **(B)** The ROC curve for the ApHCM diagnosis analyses in △T10^th^−90^th^. (△T10^th^−90^th^ = T-wave-10^th^ – T-wave-90^th^; △T_*HRmax–HRmi*_*_*n*_* = T_*HRmax*_ – T_*HRmin*_).

To test the influence of the different T-waves’ percentiles on detection performance, the T-waves of the 10^th^, 50^th^, and 90^th^ percentiles were compared with the 267 ECG data of the CAD. In Figure 3A, we calculated the receiver operating characteristic curve (ROC), which is used to analyze different T-waves percentages. However, when the T-wave-90^th^ compared with T-wave-10^th^ and T-wave-50^th^, detection performance significantly improved. In Figure 3B, the ROC of △T10^th^−90^th^ was 0.923, suggesting that the amplitude of the T-wave change is also important to distinguish ApHCM from CAD. Additionally, the T-wave-90^th^ achieved an average sensitivity and specificity of 0.903 and 0.921, which are significantly higher than those of the T-wave-10^th^ and T-wave-50^th^. When △T10^th^−90^th^ is more than 3.35 mm, average sensitivity and specificity of detection performances of 0.819 and 0.899 were achieved.

### The distribution of T-wave-max corresponds to the patient at different ages

**FIGURE 4:**
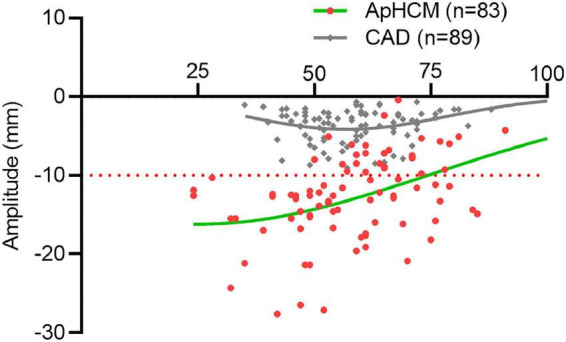
The distribution of T-wave-max corresponds to patients at different ages. The amplitudes of T-wave-max are taken as ordinate and the age of the patient as abscissa. The green and gray plots represent the ApHCM and CAD groups, respectively, while the curve shows the trend of T-wave-max amplitudes.

T-wave-max amplitudes were >10 mm in 57 (68%) patients with ApHCM but <10 mm in all patients in the CAD group (Figure 4). In patients with ApHCM who were aged <50 years (*n* = 24, 100%), all T-wave-max amplitudes were >10 mm. In the 33 (55.9%) patients with ApHCM who were aged >50 years, T-wave-max amplitudes were >10 mm. For patients over 50 years of age in the ApHCM group, T-wave-max amplitude decreased with age.

## Discussion

Long-term T-wave changes in ApHCM have been widely recognized by cardiologists. From the initial appearance of hypertrophy and deepened T-waves and after a certain period, T-waves gradually become shallow, displaying a U-shape (5). However, our study found that T-wave depth was unstable over the course of 24 h, and the change in T-waves in patients with ApHCM was more obvious than that in patients with CAD. In the short term, various factors (such as heart rate and circadian rhythm) were shown to affect the amplitude of inverted T-waves in ApHCM.

The common symptoms of patients with HCM are chest pain, dyspnea, palpitations, and syncope (19–21), and these symptoms overlap with those in patients with CAD. The symptoms of patients with ApHCM, a specific type of HCM, are similar to those of HCM and need to be differentiated from CAD. The amplitude of deep inverted T-waves in the ApHCM and CAD groups negatively correlated with heart rate, while the change in T-waves in the ApHCM group was more obvious than that in the CAD group. However, the slowest heart rate did not correspond to the deepest T-wave, and the fastest heart rate did not correspond to the shallowest T-wave. Other factors also affected T-wave amplitude. Our study found that the average T-wave amplitude from 23:00 to 07:00 was obviously different from that during the rest of the day, indicating that the degree of T-wave inversion changed between day and night, with deeper T-waves more likely to occur at night. The circadian rhythm of ApHCM group was more obvious than that of CAD group.

The mechanism underlying the change in T-waves over 24 h may be that of the “normalization of abnormal T-waves effect” (NATWE). In stress test, the characteristics of fast heart rate and shallow T-waves are similar to that of the NATWE (22). The NATWE is observed in CAD, myocarditis, cardiomyopathy, hypertension, and premature repolarization syndrome (23). The mechanism of NATWE involves myocardial ischemia and hypoxia leading to a massive release of potassium ions from myocardial cells, resulting in temporary hyperkalemia and thus, abnormal ventricular repolarization (24, 25). Intense exercise-induced ventricular remodeling is a potential adaptation of cardiac function and structure (26, 27), and NATWE may also be involved in the process of ventricular remodeling in patients with ApHCM. The positive amplitude of the T-wave (which is inverted and added to the previous T-wave) thus increases, resulting in the normalization or “swallowing” of the inverted T-wave both in ApHCM and CAD. Moreover, the influence of NATWE in ApHCM is significantly greater than that in CAD. Deeper T-waves at night relate to the energy metabolism of myocardium, which is lower and less influenced by NATWE at night.

Our study found significant changes in T-waves over the 24-h period, with deeper T-waves occurring more frequently at night. This observation may be due to the higher probability of the patient lying down in the supine position at night, considering the effect of body position on T-waves. However, we found that changes in T-waves at night were still present. In addition, 49 of the 83 patients who underwent two or more routine 12-lead ECGs showed significant changes in T-wave amplitude, but no changes in posture, among their multiple routine ECGs (Supplementary Table 1). This indicates that body position does not affect T-wave amplitude.

Coronary artery disease is one of the most important risk factors for cardiovascular events in patients with ApHCM (28). It can cause myocardial ischemia and hypoxia, which often affects T-wave amplitudes (29). However, analyses of the ApHCM subgroups showed that no significant difference in inverted T-wave amplitude existed between patients with ApHCM who did or did not have CAD. This result requires further evaluation of the changes in T-waves at the exact time of myocardial ischemia in CAD.

No authoritative, ApHCM-specific recommendations to guide ApHCM diagnosis currently exist (7). Detecting the amplitude of T-waves in patients with ApHCM may affect the diagnostic ability of ECG (9). The evaluation test of several cardiologists confirmed that inverted T-wave amplitude >10 mm significantly improved the diagnosis of ApHCM and that the diagnosis of 46% of patients with ApHCM was affected by the change in T-waves. In our study, T-wave of patients with ApHCM changed significantly in a short amount of time, and these changes may have been caused by NATWE. It is extremely important to find ECG fragments that are less affected by NATWE. An ECG performed in an inactive state (comparable to sleep) or finding deeper T-waves in DCG may facilitate finding ECG fragments that are less affected by NATWE. Comparing the T-wave-max amplitude of the ApHCM and CAD groups, T-wave-max amplitudes were >10 mm in 68% of patients with ApHCM, but <10 mm in all patients in the CAD group. T-wave-max amplitudes in patients with ApHCM aged <50 years (approximate intermediate follow-up group or earlier) were all >10 mm. T-wave amplitudes in patients aged >50 years decreased significantly (approximate long-term follow-up group). Therefore, DCG may dramatically improve the specificity and sensitivity of ApHCM in the early and middle stages, by detecting inverted T-wave amplitudes above 10 mm.

Through DCG, we know that remarkable changes in T-waves occur in ApHCM: (1) The change in T-waves in patients with ApHCM was significantly greater than that in patients with CAD; (2) degree of T-wave inversion changes diurnally, with deeper inverted T-waves occurring more frequently at night; and (3) inverted T-wave amplitude has a specific correlation with heart rate, where deeper inverted T-waves are more likely to appear in slower heart rates. At present, there are many machine learning models for identifying the phenotypes of HCM by ECG (30, 31), and the artificial intelligence detection method for ApHCM should be further explored.

## Limitations

This study has some limitations. This study was a retrospective analysis, excluding patients with ApHCM who did not undergo coronary angiography, which may have exaggerated the proportion of patients with concurrent CAD; thus, the clinical application value of our findings needs to be further verified by prospective studies.

## Conclusion

Our study showed striking variations in the T-waves in patients with ApHCM compared with patients with CAD, over the course of 24 h. The amplitude of inverted T-waves was related to heart rate and circadian rhythm, and T-wave changes in ApHCM may be due to the NATWE. The amplitude of T-waves affects the diagnosis of ApHCM, and finding the ECG fragments of inverted T-waves that are less influenced by the NATWE will help improve the diagnostic rate of ApHCM—especially for patients with ApHCM aged <50 years. ECG examinations during a state of inactivity (comparable to sleep) or 24-h DCG examinations will allow for a more sensitive diagnosis of ApHCM.

## Data availability statement

The raw data supporting the conclusions of this article will be made available by the authors, without undue reservation.

## Ethics statement

The studies involving human participants were reviewed and approved by the Tongji Medical College, Huazhong University of Science and Technology. Written informed consent for participation was not required for this study in accordance with the national legislation and the institutional requirements.

## Author contributions

FM conceived the project, participated in the writing, and created the figures. YY, JT, XD, XC, XB, and TD were responsible for collecting the data, preprocessing, and annotating data sets. XY and SL put forward constructive suggestions for revision. FL designed the idea for the manuscript, led the whole project, and made a strict evaluation of the manuscript. All authors contributed to the article and approved the submitted manuscript.
