# Supplementary material for: Twenty-four hour variability of inverted T-waves in patients with apical hypertrophic cardiomyopathy
Source: Front Cardiovasc Med. 2022 Sep 21;9:1004178. doi: 10.3389/fcvm.2022.1004178 (PMC9532612; doi:10.3389/fcvm.2022.1004178)
Supplement: Supplementary file 1 [file Data_Sheet_1.pdf]

Supplement

T wave difference of 12-lead standard ECG in same patient at different times.

49 of 83 patients underwent two or more routine 12-lead ECGs in two weeks. Therefore, we analyzed the T waves of 12-lead resting ECG of the same patient at different times (**Supplement Table 1**). There were 17 patients (34.69%) whose variation in T wave amplitude was less than 3 mm, 12 patients (24.49%) whose variation was greater than 6 mm, and 20 patients (40.82%) whose variation was between 3 and 6 mm.

Supplement Table 1. Difference in T wave on 12-lead standard ECG in the same patient at different times

T-wave variation (mm)	N (49)
0-3	17 (34.69%)
3-6	20 (40.82%)
6-9	10 (20.41%)
>9	2 (4.08%)

Analysis of T-wave distribution characteristics in patients with CAD during different periods of the day

The mean T-wave amplitude in the first and second day-time periods was -2.05 ± 1.45 mm and -2.10 ± 1.52 mm, respectively; the differences between the two time periods were not statistically significant. However, the mean T-wave amplitude in the third day-time period was -2.38 ± 1.61 mm, which was also different from that in the first and second day-time periods (p-value < 0.001; Supplementary Figure 1a).

We calculated ECG distribution probabilities corresponding to the 10th, 50th, and 90th percentile T-waves for the different day-time periods. The distribution characteristics of T-wave in patients with CAD during different periods of the day also have a certain circadian rhythm (Supplementary Figure 1b), but the degree is not as obvious as that of ApHCM.

Supplementary Figure 1

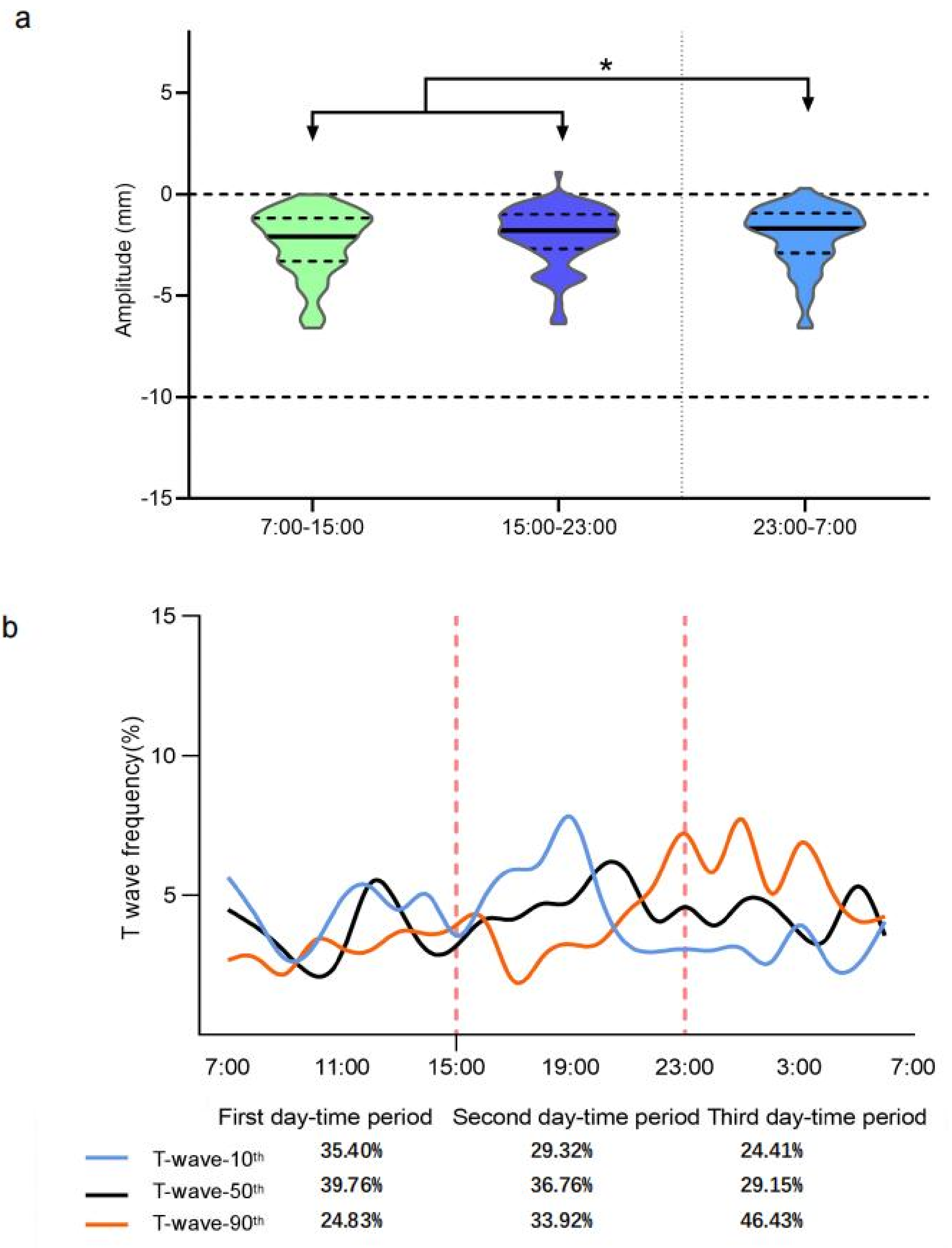

Supplementary Figure 1.**Analysis of T-wave distribution characteristics in patients with CAD at different periods of the day.** The average T-wave amplitude from 07:00 to 15:00 (the first day-time period) was -2.05 ± 1.45 mm and that from 15:00 to 23:00 (the second day-time period) hours was -2.09 ± 1.51 mm; the amplitude of T-waves were similar in the first and second day-time periods. The average T-wave amplitude from 15:00 to 23:00 (the third day-time period) was -2.38 ± 1.61 mm, which was slightly increased from the T-wave amplitudes recorded in the first and second day-time periods (*$p<0.01$; **a**). The distribution probabilities of ECGs corresponding to the 10th, 50th, and 90th percentile T-waves in different time periods of a day (**b**).
